# An unexpected role of neuroligin-2 in regulating KCC2 and GABA functional switch

**DOI:** 10.1186/1756-6606-6-23

**Published:** 2013-05-12

**Authors:** Chicheng Sun, Lei Zhang, Gong Chen

**Affiliations:** 1 Department of Biology, The Huck Institutes of Life Sciences, The Pennsylvania State University, University Park, PA 16802, USA

**Keywords:** Neuroligin-2, KCC2, GABA, Intracellular chloride homeostasis, Synapse formation, Excitation-inhibition balance

## Abstract

**Background:**

GABA_A_ receptors are ligand-gated Cl^-^ channels, and the intracellular Cl^-^ concentration governs whether GABA function is excitatory or inhibitory. During early brain development, GABA undergoes functional switch from excitation to inhibition: GABA depolarizes immature neurons but hyperpolarizes mature neurons due to a developmental decrease of intracellular Cl^-^ concentration. This GABA functional switch is mainly mediated by the up-regulation of KCC2, a potassium-chloride cotransporter that pumps Cl^-^ outside neurons. However, the upstream factor that regulates KCC2 expression is unclear.

**Results:**

We report here that KCC2 is unexpectedly regulated by neuroligin-2 (NL2), a cell adhesion molecule specifically localized at GABAergic synapses. The expression of NL2 precedes that of KCC2 in early postnatal development. Upon knockdown of NL2, the expression level of KCC2 is significantly decreased, and GABA functional switch is significantly delayed during early development. Overexpression of shRNA-proof NL2 rescues both KCC2 reduction and delayed GABA functional switch induced by NL2 shRNAs. Moreover, NL2 appears to be required to maintain GABA inhibitory function even in mature neurons, because knockdown NL2 reverses GABA action to excitatory. Gramicidin-perforated patch clamp recordings confirm that NL2 directly regulates the GABA equilibrium potential. We further demonstrate that knockdown of NL2 decreases dendritic spines through down-regulating KCC2.

**Conclusions:**

Our data suggest that in addition to its conventional role as a cell adhesion molecule to regulate GABAergic synaptogenesis, NL2 also regulates KCC2 to modulate GABA functional switch and even glutamatergic synapses. Therefore, NL2 may serve as a master regulator in balancing excitation and inhibition in the brain.

## Background

A delicate balance between excitatory and inhibitory neurotransmission is critical for brain functions. GABA is the principle inhibitory neurotransmitter in the adult brain, and dysfunction of GABAergic transmission may contribute to the onset of many neurological disorders including epilepsy, schizophrenia, autism spectrum disorders, and major depressive disorders [1-3]. Because GABA_A_ receptors are ligand-gated Cl^-^ channels, the efficacy of GABAergic transmission is modulated by changes in intracellular Cl^-^ concentration. Two chloride transporters, NKCC1 and KCC2, import and export Cl^-^ across neuronal membranes correspondingly. In early neural development, the expression level of NKCC1 is initially high and gradually down-regulated, while KCC2 expression is up-regulated [4,5]. Such developmental changes of NKCC1 and KCC2 result in a shift of intracellular Cl^-^ concentration from high to low and a corresponding shift of GABA_A_ receptor reversal potential from depolarizing to hyperpolarizing [6,7]. Therefore, GABA is not a simple inhibitory neurotransmitter, but rather undergoes a functional switch from excitation to inhibition during brain development. GABA-mediated excitation regulates neural differentiation, migration, and synaptogenesis [4,8-10]. Our previous work found that GABAergic synaptogenesis precedes glutamatergic synaptogenesis due to the earlier expression of GABA_A_ receptors than that of glutamate receptors in embryonic neurons [11]. So far, it is unclear whether there is any coordination between GABA functional switch and GABAergic synapse formation.

Neuroligins (NLs) are a family of postsynaptic cell adhesion molecules that interact with presynaptic neurexins [12]. NL1 and NL2 are selectively localized at glutamatergic and GABAergic synapses [13,14], and manipulations of NL1 and NL2 expression level have been shown to regulate glutamatergic and GABAergic synaptogenesis, respectively [15-17]. Transgenic mice overexpressing NL2 showed enhanced GABAergic transmission [18], whereas NL2 knockout mice showed decreased GABAergic transmission [19]. We have previously shown that NL2 is a critical cell adhesion molecule capable of inducing functional GABAergic synapses in neuron-HEK cell hetero-cocultures [20]. Our recent work further identified a loss-of-function mutation of NL2 in schizophrenia patients [21], suggesting an indispensable role of NL2 in regulating GABAergic functions.

Here, we uncover a novel function of NL2 in regulating KCC2 expression and GABA functional switch from excitation to inhibition during neurodevelopment. Knockdown of NL2 also induces an unexpected reduction in glutamatergic events and dendritic spines. Therefore, in addition to its role as a cell adhesion molecule at GABAergic synapses, NL2 may serve as a master regulator of the delicate balance between glutamatergic and GABAergic functions in neural networks.

## Results

### Neuroligin-2 unexpectedly regulates KCC2 expression

**Figure 1:**
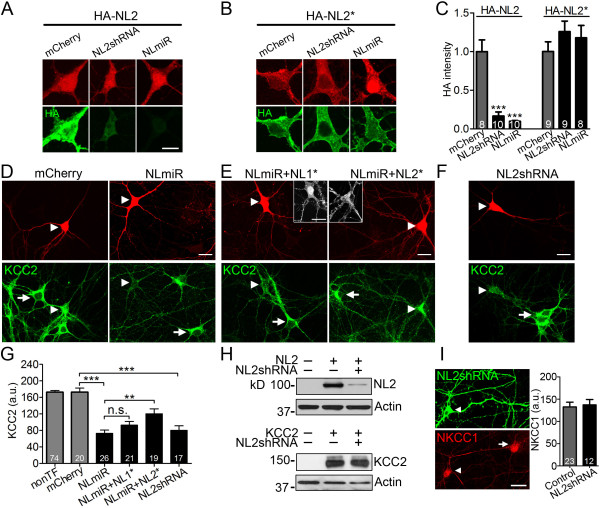
**Knockdown of neuroligin-2 decreases KCC2 expression. *A***, Efficient knockdown of NL2 by NL2shRNA or NLmiR in mouse cortical neurons. Neurons were co-transfected with mCherry at 2 DIV and HA-NL2 expression was assayed by HA-immunostaining at 8 DIV. ***B***, shRNA-proof mutant HA-NL2* was resistant to the knockdown of NL2shRNA or NLmiR. Scale bar, 10 μm. ***C***, Bar graphs showing quantified somatic HA-immunostaining intensity (normalized by mCherry controls). ***p < 0.001 (one-way ANOVA). ***D***, Representative images showing reduced KCC2 immunostaining (green) in NLmiR-transfected neurons (arrowhead) compared to non-transfected neurons (arrow, 12 DIV). Scale bar, 20 μm. ***E***, NL2*, but not NL1* rescued the KCC2 level when coexpressed with NLmiR. Inlets showed HA-immunostaining (gray) to confirm the expression of HA-NL1* and HA-NL2*. Scale bar, 20 μm. ***F***, NL2shRNA also decreased KCC2 expression level. Scale bar, 20 μm. ***G***, Bar graphs showing quantified somatic KCC2 immunofluorescence intensity in non-transfected (nonTF) neurons (173 ± 4 a.u.) and neurons transfected with mCherry (172 ± 10), NLmiR (73 ± 8), NLmiR + NL1* (93 ± 9), NLmiR + NL2* (120 ± 12), or NL2shRNA (80 ± 11). a.u., arbitrary unit. **p < 0.01, ***p < 0.001, n.s., not significant, one-way ANOVA. ***H***, NL2shRNA specifically knock down NL2 but not KCC2 in HEK 293T cells, which were co-transfected with NL2 + GFP, NL2 + NL2shRNA, KCC2 + GFP, or KCC2 + NL2shRNA. Total protein lysate was analyzed by immunoblot. Actin was used as loading control. ***I***, NKCC1 immunostaining signal (red) was not altered in NL2shRNA-transfected neurons (green, arrowhead, 9 DIV). Scale bar, 20 μm. Bar graphs show the quantified somatic NKCC1 signal intensity (*p* > 0.7, unpaired Student’s *t*-test).

Neuroligin-2 (NL2) is a cell adhesion molecule mainly localized at GABAergic synapses [14]. We have previously demonstrated that overexpression of NL2 and GABA_A_ receptors in HEK cells can induce fully functional GABAergic synapses when cocultured with neurons [20]. Recently we also identified a mutant NL2 from human schizophrenia patient that is defective in promoting GABAergic synapse formation [21]. During our continued study of NL2 in synapse formation and plasticity, we made an unexpected finding that NL2 regulates KCC2, a K^+^-Cl^-^ cotransporter that is critical in controlling intracellular Cl^-^ concentration and the polarity of GABA action. We investigated the function of NL2 by using small-hairpin RNA (shRNA) mediated knockdown in cultured mouse cortical neurons. Two previously well characterized shRNAs were used to knockdown NL2 expression level: one is a chained shRNA targeting all NL1-3 (NLmiR) [22] and the other is a NL2-specific shRNA (NL2shRNA) [15]. When HA-tagged wild type (WT) NL2 was coexpressed with NL2shRNA or NLmiR, the expression level of HA-NL2 was reduced by more than 80% compared to the coexpression with mCherry alone as a control (Figure 1A, quantified in Figure 1C left columns). In contrast, the expression of a shRNA-proof mutant version of NL2 (HA-NL2*) [22] was not affected by NL2shRNA or NLmiR (Figure 1B, quantified in Figure 1C right columns). Therefore, both NLmiR and NL2shRNA can efficiently knockdown NL2, and the mutant NL2* was suitable for rescue experiments.

When we transfected mouse cortical neurons with NLmiR, we observed a significant reduction of the KCC2 expression level compared to that of non-transfected (nonTF) or mCherry-transfected control neurons (Figure 1D, quantified in Figure 1G). This is unexpected because no previous study reported any connection between NL2 and KCC2. To find out the relative contribution of NL1 versus NL2 to the reduced KCC2 expression, shRNA-proof HA-NL1* or HA-NL2* was coexpressed with NLmiR to test which one can rescue the KCC2 expression. HA-immunostaining confirmed the expression of shRNA-proof NL1* and NL2* in the presence of NLmiR (Figure 1E inlets). Coexpression of HA-NL2*, but not HA-NL1*, with NLmiR significantly rescued the KCC2 expression level (Figure 1E, quantified in Figure 1G), suggesting that NL2 is a potential regulator of KCC2. This was confirmed by the detection of a similar reduction of KCC2 level after knocking down NL2 specifically with NL2shRNA (Figure 1F, quantified in Figure 1G). One concern regarding shRNAs is whether they might have off-target effect directly on KCC2. To exclude this possibility, we co-transfected NL2shRNA together with KCC2 in HEK 293T cells to examine whether KCC2 expression might be altered. Immunoblot demonstrated that while WT NL2 expression was significantly reduced by NL2shRNA (Figure 1H, top panel), KCC2 expression was not affected by NL2shRNA (Figure 1H, bottom panel). We next examined the effect of NL2shRNA on NKCC1, which imports Cl^-^ and counteracts the action of KCC2 [7]. Immunostaining with antibody specific for NKCC1 showed that NL2 knockdown did not change NKCC1 expression level (Figure 1I). Together, our data demonstrated a novel function of NL2 in regulating KCC2, both of which were found previously playing critical roles in GABA function but not yet linked together.

### Neuroligin-2 regulates GABA excitation-inhibition switch

**Figure 2:**
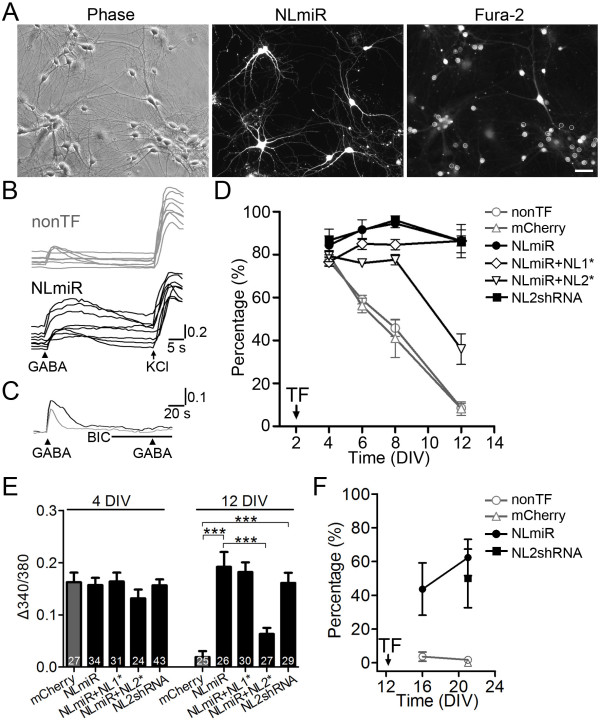
**Knockdown of neuroligin-2 abolishes GABA functional switch in cortical neurons. *A***, Representative images showing 8 DIV NLmiR-transfected neurons loaded with Fura-2. Somata of both nonTF and transfected neurons were selected (Fura-2 panel, white circles) to measure 340/380 ratio signal. Scale bar, 40 μm. ***B***, Sample traces showing somatic Ca^2+ ^responses after stimulation with 100 μM GABA and 90 mM KCl in nonTF neurons (gray, 8 DIV) and NLmiR-transfected neurons (black). ***C***, Averaged sample traces showing that BIC (20 μM) blocked GABA-evoked Ca^2+^ responses in both nonTF (gray, *n *= 6) and NLmiR-transfected neurons (black, *n *= 17). ***D***, The time courses of GABA functional switch in nonTF neurons and neurons transfected with mCherry, NLmiR, NLmiR + NL1*, NLmiR + NL2*, or NL2shRNA (3–4 independent cultures; *n *= 2291, 272, 433, 350, 322, 152 neurons, respectively). ***E***, Bar graphs showing the amplitude of GABA-evoked Ca^2+ ^increases in neurons transfected with mCherry, NLmiR, NLmiR + NL1*, NLmiR + NL2*, or NL2shRNA at 4 and 12 DIV. There was no difference between all groups at 4 DIV (*p *> 0.6). ***p < 0.001, one-way ANOVA. ***F***, Percentage of mature neurons showing GABA-evoked Ca^2+ ^increase after NLmiR or NL2shRNA transfection. Arrows in ***D ***and ***F ***indicate the time of transfection (TF).

KCC2 is a key player in controlling intracellular Cl^-^ concentration and driving GABA functional switch from excitation to inhibition during early development [23,24]. Therefore, if KCC2 is regulated by NL2, we predict that GABA functional switch might be affected accordingly. To test this hypothesis, cortical neurons were co-transfected at 2 DIV by mCherry with NLmiR, and Fura-2 Ca^2+^ ratio imaging was employed to monitor GABA-evoked Ca^2+^ influx to determine whether GABA action is excitatory or inhibitory [25]. When neurons were analyzed 6 days later (at 8 DIV), we found that GABA (100 μM) application evoked small Ca^2+^ responses in less than 50% of non-transfected control neurons but large Ca^2+^ responses in over 90% of NLmiR-transfected neurons (Figure 2A-B). As a control, high potassium (90 mM) stimulation induced robust Ca^2+^ responses in both non-transfected and NLmiR-transfected neurons (Figure 2B). GABA-evoked Ca^2+^ increase was completely blocked by GABA_A_ receptor antagonist bicuculline (BIC, 20 μM) (Figure 2C), suggesting that Ca^2+^ influx was mediated by GABA_A_ receptor activation.

Using Ca^2+^ imaging approach, we further delineated the time course of GABA functional switch by monitoring the gradual decrease of GABA-evoked Ca^2+^ responses in developing neurons. We found that mouse cortical neurons typically complete their GABA functional switch around two weeks in dissociated cultures (Figure 2D), similar to previous reports [26,27]. Quantitatively, for control neurons transfected with mCherry, GABA evoked Ca^2+^ responses in 80 ± 5% (*n* = 96) neurons at 4 DIV, but only 8 ± 3% (*n* = 61) at 12 DIV (Figure 2D), suggesting that the majority of neurons have finished the GABA excitation-inhibition transition by 12 DIV. However, after NL knockdown, even at 12 DIV, GABA still evoked Ca^2+^ responses in more than 80% of transfected neurons (Figure 2D; NLmiR, 86 ± 5%, *n* = 86; NL2shRNA, 86 ± 8%, *n =* 41; *p* < 0.001 for transfection of mCherry vs. NLmiR or NL2shRNA, two-way ANOVA). Importantly, coexpression of NLmiR with NL2*, but not NL1*, promoted GABA functional switch by 12 DIV (Figure 2D; NLmiR vs. NLmiR+NL2*, *p* < 0.001, one-way ANOVA at 12 DIV), suggesting that NL2 may regulate GABA excitation-inhibition switch.

Besides quantifying the percentage of neurons responding to GABA, we also compared the amplitude of GABA-evoked Ca^2+^ responses in individual neurons. At 4 DIV, most neurons in all groups showed significant GABA-evoked Ca^2+^ responses, suggesting an excitatory action of GABA (Figure 2E). At 12 DIV, while control neurons transfected with mCherry showed very small Ca^2+^ responses, neurons transfected with NLmiR or NL2shRNA still showed large GABA-evoked Ca^2+^ responses with the amplitudes similar to those at 4 DIV (Figure 2E), suggesting no GABA functional switch occurred after knockdown of NL2. Coexpression of NLmiR with NL2*, but not NL1*, resulted in significantly smaller GABA-evoked Ca^2+^ responses at 12 DIV (Figure 2E), suggesting that NL2 may restore GABA functional switch. Collectively, our Ca^2+^ imaging data demonstrate that NL2 plays a critical role in regulating GABA functional switch during early development.

We further investigated whether NL2 is required for maintaining GABAergic inhibition in mature neurons after the completion of GABA functional switch. To address this question, we transfected neurons at 12–14 DIV with NLmiR or NL2shRNA and analyzed GABA-evoked Ca^2+^ responses at 16 and 21 DIV. Mature neurons in control group rarely showed any GABA-evoked Ca^2+^ responses (Figure 2F; non-transfected, only 4 out 264 neurons; mCherry-transfected, 0/7), but more than 50% mature neurons transfected with NLmiR (*n* = 59) or NL2shRNA (*n* = 12) showed significant GABA-evoked Ca^2+^ responses (Figure 2F). Therefore, NL2 is not only required for GABA functional switch in immature neurons, but also required for the maintenance of GABA inhibition in mature neurons.

### Neuroligin-2 regulates GABA equilibrium potential

**Figure 3:**
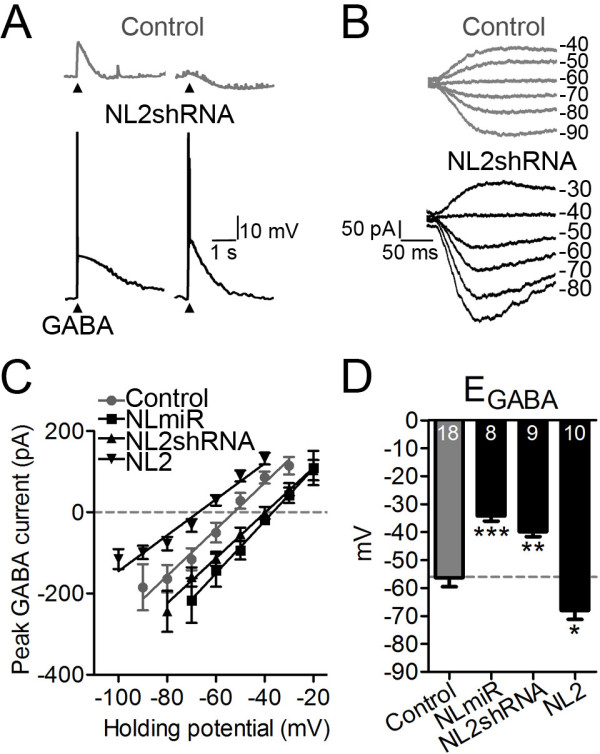
**Neuroligin-2 regulates GABA equilibrium potential. *A***, Representative current clamp recordings showing differential changes in membrane potentials evoked by GABA in 10–13 DIV control neurons (gray) and NL2shRNA-transfected neurons (black). Note that GABA evoked action potentials in most NL2shRNA-transfected neurons (4 out of 5 neurons) but rarely in control neurons (1/6). ***B***, Representative voltage-clamp recordings showing GABA-evoked currents at different holding potentials in the presence of TTX (0.5 μM) and DNQX (10 μM). ***C***, I-V plot of the mean GABA-evoked peak currents in control neurons and neurons transfected with NLmiR, NL2shRNA, or NL2. Note the opposite shift in E_GABA_ between NL2 overexpression and NL2 knockdown groups. ***D***, Bar graphs showing mean E_GABA _of control neurons and neurons transfected with NLmiR, NL2shRNA, or NL2. Dashed line indicates the mean E_GABA_ of control neurons. *p < 0.05, **p < 0.01, ***p < 0.001, one-way ANOVA.

The observed KCC2 reduction and large GABA-evoked Ca^2+^ responses after NL2 knockdown suggest an excitatory action of GABA due to depolarized GABA_A_ receptor reversal potential (E_GABA_). To directly examine E_GABA_, we performed gramicidin-perforated patch clamp recordings to keep intracellular Cl^-^ intact [26]. In control neurons at 10–13 DIV, GABA application (40 μM, 50 ms) typically evoked small depolarizing or hyperpolarizing membrane potential changes (Figure 3A, top traces). In contrast, in neurons transfected with NL2shRNA, GABA reliably evoked action potentials on top of large depolarizing responses (Figure 3A, bottom traces), confirming that GABA function remains excitatory after NL2 knockdown. Changing holding membrane potentials under voltage-clamp condition revealed a significant depolarizing shift in E_GABA_ after NL2 knockdown (Figure 3B). Quantitatively, knockdown of NL2 alone resulted in a depolarizing shift of 16 mV in E_GABA_ and knockdown of NL1-3 induced a shift of 22 mV (Figure 3C-D; E_GABA_: Control, -56 ± 3 mV; NL2shRNA, -40 ± 2 mV; NLmiR, -34 ± 2 mV). Interestingly, overexpression of NL2 caused an opposite change: a significant hyperpolarizing shift of 12 mV in E_GABA_ (Figure 3C-D; NL2, -68 ± 3 mV). On the other hand, the resting membrane potential was not significantly altered by NL2 manipulations (Control, -60 ± 2 mV; NLmiR, -58 ± 4 mV; NL2shRNA, -63 ± 2 mV; NL2, -67 ± 2 mV; *p* > 0.08). These results suggest that NL2 plays an active role in controlling the functional polarity of GABA action.

### Regulation of KCC2 by neuroligin-2 is independent of GABA_A_ receptor activation and neuronal activity

**Figure 4:**
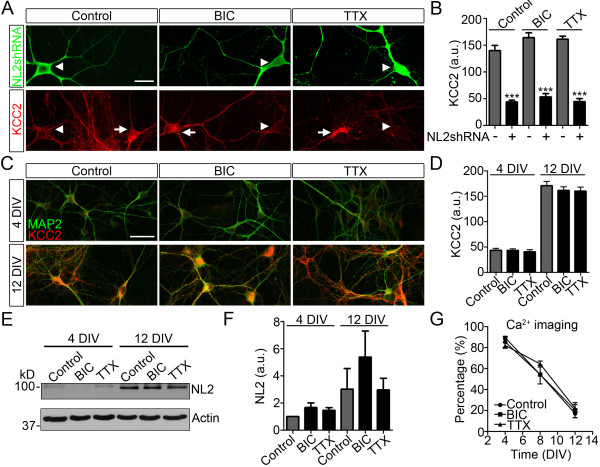
**Regulation of KCC2 by NL2 is independent of GABA**_**A **_**receptor function or neuronal activity. *A***, Representative images showing reduced KCC2 immunostaining (red) in neurons transfected with NL2shRNA (green, arrowheads, 11 DIV), regardless whether treated with BIC or TTX. DMSO (0.1%, Control), BIC (20 μM) or TTX (1 μM) was added into culture medium after transfection at 2 DIV and replenished every two days. Scale bar, 20 μm. ***B***, Bar graphs showing somatic KCC2 immunofluorescence intensity in neurons with (+) and without (-) NL2shRNA transfection (*n *= 11–12 neurons per group). Cultures were treated with DMSO (Control), BIC or TTX after transfection. ***p < 0.001, unpaired Student’s *t*-test. ***C***, Representative images showing MAP2 (green) and KCC2 (red) immunostaining at 4 and 12 DIV in neurons treated with DMSO, BIC or TTX, starting at 2 DIV. Scale bar, 20 μm. ***D***, Bar graphs showing developmental increase of the somatic KCC2 immunofluorescence intensity (*n *= 12 neurons per group). a.u., arbitrary unit. ***E***, Representative immunoblot showing NL2 expression in 4 and 12 DIV neurons treated with DMSO, BIC or TTX, starting at 2 DIV. Actin was used as loading control. ***F***, Quantification of NL2 expression level as measured by immunoblot (*n *= 3 independent cultures). NL2 expression was normalized to the expression level of 4 DIV control. ***G***, Calcium imaging showing the time courses of GABA functional switch in control neurons and neurons treated with BIC or TTX (3 independent cultures; *n* = 1141, 717, 737 neurons, respectively).

Since NL2 regulates GABAergic synaptogenesis, we wondered whether the regulation of KCC2 by NL2 might be mediated by an alteration in GABA_A_ receptor activation or neuronal activity. To test this idea, cortical neurons were transfected with NL2shRNA at 2 DIV and chronically treated for 9 days with BIC (20 μM) to block GABA_A_ receptors or with tetrodotoxin (TTX, 1 μM) to block action potential firing. Blocking the activation of GABA_A_ receptors appeared to have no effect on the KCC2 level because the neurons transfected with NL2shRNA still showed a significant reduction of KCC2 compared to adjacent non-transfected controls (Figure 4A middle panel, quantified in Figure 4B). Similarly, inhibiting action potential firing also had no effect on the reduction of KCC2 induced by NL2 knockdown (Figure 4A right panel, quantified in Figure 4B). Therefore, NL2 regulation of KCC2 is not mediated by GABA_A_ receptors or neuronal activity.

We then investigated whether developmental expression of KCC2 and NL2 rely upon GABA_A_ receptor activation or neuronal activity. As expected, KCC2 expression showed a significant increase from 4 to 12 DIV (Figure 4C left panel). After chronic treatment with BIC or TTX, KCC2 level was similarly increased compared to the control (Figure 4C middle and right panels, quantified in Figure 4D; *n* =12 neurons per group; *p* < 0.001 for developmental increase; *p* > 0.5 for drug treatment, Two-way ANOVA). Immunoblot analysis also found that NL2 expression level increased significantly from 4 to 12 DIV and were not affected by BIC or TTX treatment (Figure 4E-F; *n* = 3; *p* < 0.05 for developmental increase; *p* > 0.3 for drug treatment, Two-way ANOVA). Functionally, Ca^2+^ imaging experiments further showed comparable time courses of GABA functional switch between control and BIC- or TTX-treated neurons (Figure 4G; *p* > 0.7 for drug treatment, Two-way ANOVA). Together, our data suggest that the developmental up-regulation of NL2 and KCC2 as well as GABA functional switch are likely regulated by cell-intrinsic mechanisms, independent of GABA_A_ receptor activation or neuronal activity.

### Neuroligin-2 expression precedes KCC2 *in vivo*

**Figure 5:**
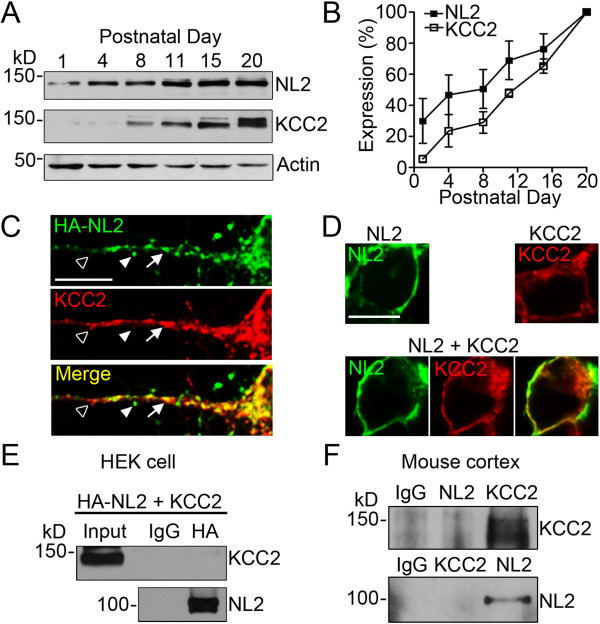
**Neuroligin-2 expression precedes that of KCC2. *A***, Representative immunoblot showing the time-course of NL2 and KCC2 expression in mouse cortex at postnatal day 1 (P1), P4, P8, P11, P15, and P20. Actin was used as loading control. ***B***, Quantification of the NL2 and KCC2 expression level as measured by immunoblot (*n *= 3). Protein expression was normalized to the expression level at P20. ***C***, Representative images showing the expression of HA-NL2 (green) and KCC2 (red) in mouse cortical neurons (14 DIV) transfected with HA-NL2. Empty arrowhead indicates KCC2 puncta without NL2 colocalization; Filled arrowhead indicates NL2 puncta without KCC2 colocalization; Arrow indicates colocalized NL2 and KCC2 puncta. Scale bar, 10 μm. ***D***, Representative images showing the expression pattern of NL2 (green), KCC2 (red), and NL2 + KCC2 in HEK cells. Scale bar, 10 μm. ***E***, Lack of co-immunoprecipitation revealed by HEK cells co-transfected with HA-NL2 and KCC2. Total protein lysate was immunoprecipitated with normal mouse IgG or mouse anti-HA antibody and immunoblotted with rabbit anti-KCC2 and anti-NL2 antibodies. ***F***, No co-immunoprecipitation detected between NL2 and KCC2 in mouse brain cortical tissue. Total mouse cerebral cortex protein lysate was immunoprecipitated with normal rabbit IgG, rabbit anti-NL2, or rabbit anti-KCC2 antibodies. The precipitated proteins were then probed with rabbit anti-KCC2 or anti-NL2 antibodies to test whether co-immunoprecipitation occurred.

We reasoned that if NL2 regulates KCC2, the onset of NL2 expression should precede that of KCC2. This was indeed what occurred in the mouse brain *in vivo* when we analyzed the time course of the expression of both NL2 and KCC2 from postnatal day 1 to day 20 (Figure 5A-B). In neonatal mouse brain (P1-P4), NL2 was already expressed in a significant amount whereas the expression of KCC2 was minimal, consistent with a delayed KCC2 expression that correlates with GABA functional switch [24]. Quantitatively, NL2 expression at P4 reached about 50% of P20 level, whereas KCC2 did not reach 50% of P20 expression level even at P11 (Figure 5B). Therefore, the *in vivo* sequential expression of NL2 and KCC2 makes it possible for NL2 to regulate KCC2.

We next investigated whether NL2 directly interacts with KCC2. Because both available NL2 (129203, Synaptic Systems) and KCC2 (07–432, Millipore) antibodies are raised in rabbit, it is not feasible to detect endogenous NL2 and KCC2 simultaneously. Instead, we overexpressed HA-NL2 in cortical neurons and found partial co-localization between exogenous HA-NL2 and endogenous KCC2 (Figure 5C). However, because HA-NL2 was overexpressed in neurons, it raised a concern whether HA immunostaining truly represents the endogenous NL2 localization. We therefore further investigated possible interaction between NL2 and KCC2 in HEK 293T cells (Figure 5D). As expected, expression of NL2 alone showed mainly membrane localization, and expression of KCC2 alone showed both membrane and intracellular localization. Coexpression of NL2 and KCC2 showed comparable subcellular localization to that of NL2 or KCC2 expression alone (Figure 5D). Next, we employed co-immunoprecipitation to examine whether NL2 and KCC2 interact with each other. Protein lysate prepared from HEK cells coexpressing HA-NL2 and KCC2 was immuno-precipitated with HA antibody. The HA-NL2 was clearly precipitated but KCC2 was not co-immunoprecipitated (Figure 5E). We further performed co-immunoprecipitation experiment using mouse cerebral cortex protein lysate and obtained similar result to HEK cells that no co-immunoprecipitation could be detected between NL2 and KCC2, regardless which protein was immuno-precipitated first (Figure 5F). These results suggest that NL2 and KCC2 may not directly interact, or their interaction is too weak to be detected with current method.

### Neuroligin-2 regulates glutamatergic transmission and dendritic spines through KCC2

**Figure 6:**
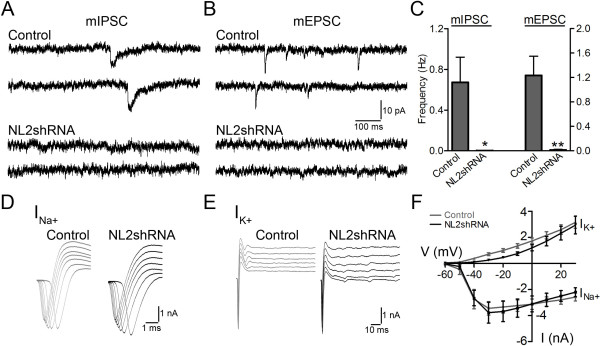
**Neuroligin-2 regulates both GABAergic and glutamatergic synapse formation. *A***-***B***, Sample traces showing mIPSCs (***A***) and mEPSCs (***B***) from 10–12 DIV control and NL2shRNA-transfected neurons. ***C***, Bar graphs showing the frequency of mIPSCs (left Y-axis) and mEPSCs (right Y-axis). *n *= 10 neurons per group, *p < 0.05, **p < 0.01, unpaired Student’s *t*-test. ***D***-***E***, Sample traces showing whole-cell sodium currents (***D***) and potassium currents (***E***) in response to depolarizing voltage steps. ***F***, I-V plot of the mean amplitude of sodium and potassium currents in control (*n* = 7) and NL2shRNA-transfected neurons (*n *= 9).

Previous study reported that knockdown of NL2 decreased not only GABAergic but also glutamatergic synapse numbers [15], but the underlying mechanism was not well understood. We transfected cortical neurons at 2 DIV with NL2shRNA and employed patch clamp recordings to analyze synaptic events at 10–12 DIV. As expected, the miniature inhibitory postsynaptic currents (mIPSCs) were largely abolished in neurons transfected with NL2shRNA (Figure 6A and C; Control, 0.67 ± 0.25 Hz, *n* = 10; NL2shRNA, 0.002 ± 0.002 Hz, *n* = 10; *p* < 0.05, unpaired Student’s *t*-test). Interestingly, the frequency of miniature excitatory postsynaptic currents (mEPSCs) was also significantly decreased (Figure 6B-C; Control, 1.2 ± 0.3 Hz, *n* = 10; NL2shRNA, 0.01 ± 0.01 Hz, *n* = 10; *p* < 0.01), confirming that NL2 not only regulates GABAergic synapses, but also affects glutamatergic synapses. We initially wondered whether the NL2 effect on glutamatergic synapses might be due to any alterations of neuronal intrinsic excitability. To test this idea, we recorded voltage-dependent sodium and potassium currents but found no difference between control and NL2shRNA-transfected neurons (Figure 6D-E). Quantitatively, the I-V curves of both Na^+^ and K^+^ currents showed no significant difference between control and NL2shRNA-transfected neurons (Figure 6F; I_Na+_, *p* > 0.9; I_K+_, *p* > 0.2, Two-way ANOVA).

**Figure 7:**
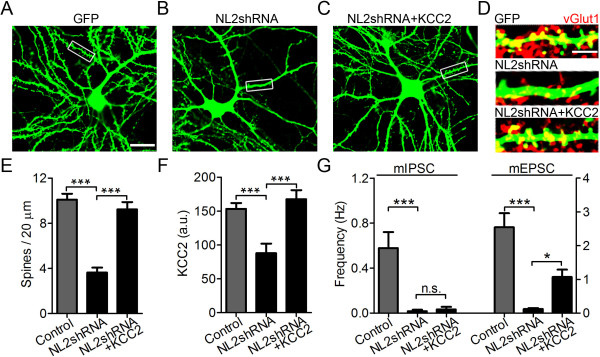
**Neuroligin-2 regulates dendritic spines through KCC2. *A***-***C***, Representative images showing dendritic spines in neurons (15 DIV) transfected with GFP (***A***), GFP-NL2shRNA (***B***), or GFP-NL2shRNA + KCC2 (***C***). Scale bar, 20 μm. ***D***, Enlarged inlets in ***A***-***C*** showing dendritic spines (green) overlaid with glutamatergic presynaptic terminals labeled by vGlut1 (red). Scale bar, 5 μm. ***E***, Quantification of dendritic spine numbers per 20 μm in neurons expressing GFP, GFP-NL2shRNA, or GFP-NL2shRNA + KCC2. *n *= 11 per group. ***F***, Bar graphs showing somatic KCC2 immunofluorescence intensity: control neurons, 153 ± 9, *n *= 17; GFP-NL2shRNA transfected neurons, 88 ± 14, *n *= 11; GFP-NL2shRNA + KCC2 transfected neurons, 168 ± 13, *n *= 9. a.u., arbitrary unit. ***G***, Bar graphs showing the frequency of mIPSCs (*n *= 9 per group; left Y-axis) and mEPSCs (*n *= 11 per group; right Y-axis). mIPSCs: Control, 0.58 ± 0.14 Hz; GFP-NL2shRNA, 0.02 ± 0.01 Hz; GFP-NL2shRNA + KCC2, 0.03 ± 0.02 Hz. mEPSCs: Control, 2.55 ± 0.42 Hz; GFP-NL2shRNA, 0.12 ± 0.03 Hz; GFP-NL2shRNA + KCC2, 1.07 ± 0.22 Hz. *p < 0.05, ***p < 0.001, n.s., not significant, one-way ANOVA.

Considering the specific targeting of NL2 to GABAergic synapses [14], the reduction in mEPSC frequency after NL2 knockdown is not easy to interpret. Since NL2 is not directly localized at glutamatergic synapses, it is possible that a different protein might mediate the effect of NL2 on glutamatergic synapses. Interestingly, KCC2 has recently been shown to modulate dendritic spines and AMPA receptor diffusion through interaction with cytoskeleton proteins [28,29]. Our above results suggest that KCC2 may be a novel downstream effector of NL2. We therefore hypothesized that NL2 may indirectly regulate glutamatergic synapses through the mediation of KCC2. To test this hypothesis, we examined the effect of NL2 and KCC2 on dendritic spine morphogenesis in mature neurons (15 DIV) (Figure 7). Consistent with the electrophysiology data, knockdown of NL2 significantly reduced dendritic spines compared to the GFP controls (Figure 7A-B). Interestingly, coexpression of KCC2 with NL2shRNA significantly rescued dendritic spines (Figure 7C). The dendritic spines were confirmed with overlaying glutamatergic presynaptic marker VGlut1 (Figure 7D). Quantitatively, the spine density in control neurons transfected with GFP alone was 10 ± 1 per 20 μm, and decreased to 4 ± 1 per 20 μm after transfection with GFP-NL2shRNA, but rescued to 9 ± 1 per 20 μm when KCC2 was coexpressed with GFP-NL2shRNA (Figure 7E). Knockdown of NL2 in mature neurons also significantly decreased KCC2 expression, and KCC2 co-transfection with NL2shRNA effectively rescued the KCC2 level (Figure 7F). Moreover, when KCC2 was coexpressed with GFP-NL2shRNA, it partially rescued the decrease of mEPSC frequency, but mIPSCs were not rescued (Figure 7G). These data suggest that NL2 directly regulates GABAergic synapse formation, while indirectly regulate glutamatergic synapse formation through KCC2.

## Discussion

**Figure 8:**
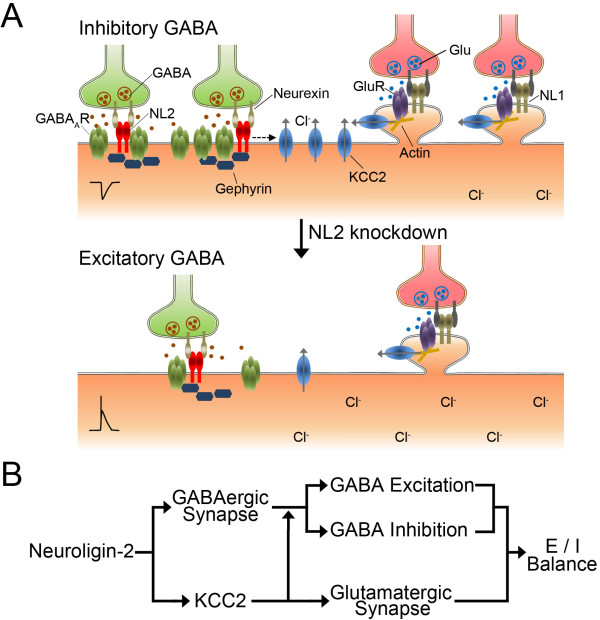
**Graphic summary of major findings and significance. *A***, Working model illustrating multiple changes induced by knockdown of NL2, including reduced GABAergic synapses, reduced glutamatergic synapses, and reduced KCC2 expression, which results in increased intracellular Cl^- ^concentration and more excitatory GABA function. ***B***, Schematic drawing illustrating NL2 as a central player in regulating the excitation and inhibition balance in the brain.

In this study, we report a novel function of neuroligin-2 in regulating KCC2 and GABA functional switch in cortical neurons. This is supported by several lines of evidences: 1) Knockdown of NL2 induced a significant decrease of the expression of KCC2, but not NKCC1; 2) Overexpressing and knockdown of NL2 caused negative and positive shift of E_GABA_, respectively; 3) After knockdown of NL2, GABA application induced large Ca^2+^ influx and even action potentials, indicating an excitatory action; 4) The decrease of KCC2 expression and GABA functional changes after knockdown of NL1-3 were rescued selectively by shRNA-proof NL2*, but not NL1*. Therefore, KCC2 is specifically regulated by NL2. We further demonstrate that NL2 may also modulate glutamatergic transmission and dendritic spines through the regulation of KCC2. Therefore, knockdown of NL2 will decrease GABAergic synapses, reduce glutamatergic synapses, and make GABA function more excitatory (summarized in Figure 8A). These novel findings support a central role of NL2 in governing the delicate balance between GABAergic and glutamatergic functions (Figure 8B).

### Classical function of neuroligin-2 in GABAergic synaptogenesis

Neuroligins and their presynaptic binding partner neurexins are important trans-synaptic cell adhesion molecules that play a critical role in synaptogenesis [12,30,31]. The synaptogenic effect of neuroligins was demonstrated by their potent induction of presynaptic differentiation when expressed in non-neuronal cells [16,20,32,33]. The NL1-3 triple knockout *in vivo* and acute NL1-3 knockdown by shRNAs *in vitro* both showed significant deficits in synaptic transmission [15,34]. It was later found that neuroligins might also play a role in synaptic validation and plasticity [35,36], possibly through a trans-synaptic feedback [37,38]. Most recent studies have revealed that NL1 may be cleaved by matrix metalloproteinases in an activity-dependent manner, which regulates glutamatergic synaptic transmission [39,40]. It will be interesting to investigate whether other NLs could also be cleaved and how the cleavage process regulates synaptic functions.

We have previously demonstrated that overexpression of NL2 with GABA_A_ receptors in HEK cells can induce fully functional GABAergic innervations from surrounding neurons [20]. We have also identified a novel loss-of-function mutation of NL2 (R215H) from human schizophrenic patient, which is incapable of inducing GABAergic innervations [21]. In the current study, we further demonstrate that knockdown of NL2 significantly reduced GABAergic synaptogenesis, consistent with previous studies [15,41]. The function of NL2 in regulating GABAergic synapse formation is likely mediated by interactions with scaffolding proteins like gephyrin and collybistin [42]. All these studies are consistent with the role of neuroligin-2 as a cell adhesion molecule to regulate GABAergic synapse formation and plasticity.

### Novel function of neuroligin-2 in regulating KCC2 and GABA functional switch

The most surprising finding of this work is the regulation of KCC2 by NL2. After knockdown of NL2, the KCC2 expression level was significantly decreased. We tested whether this might be caused by an off-target effect of the shRNAs, but coexpression of NL2shRNA with KCC2 in HEK cells showed no effect on the expression of KCC2 at all. Moreover, the shRNA-proof NL2*, but not NL1*, rescued the KCC2 expression, suggesting a NL2-specific regulation of KCC2. Similar to previously reported knockdown of KCC2 [24], we discovered that the GABA reversal potential was shifted to more depolarized level after NL2 knockdown. Moreover, after NL2 knockdown, bath application of GABA induced large Ca^2+^ influx and even triggered action potentials. These results discover a novel function of NL2 in regulating KCC2 and GABA functional switch. The regulation of KCC2 by NL2 suggests that the time-course of GABAergic synaptogenesis and GABA functional switch are tightly coordinated by NL2.

KCC2 is a Cl^-^ transporter with a major function in controlling intracellular Cl^-^ concentration and therefore playing an important role in determining whether GABA function is excitatory or inhibitory [43]. The regulation of KCC2 by NL2 greatly expands the function of NL2 beyond its classical role as a cell adhesion molecule. Since both NL2 and KCC2 are transmembrane proteins, we investigated whether NL2 directly interacts with KCC2. Our co-immunoprecipitation experiments in HEK cells and mouse brain lysate suggest a lack of direct interaction between NL2 and KCC2. However, we cannot exclude the possibility that NL2 may indirectly interact with KCC2 through other mediating proteins, such as gephyrin or GABA_A_ receptors, since overexpressed HA-NL2 showed partial colocalization with KCC2. NL2 may also regulate KCC2 activity through a variety of pathways, such as transcriptional regulation, phosphorylation, membrane trafficking, and oligomerization [44].

Since both NL2 and KCC2 regulate GABA functions, we tested whether the regulation of KCC2 by NL2 may be dependent upon GABA_A_ receptor activation. Our pharmacological experiments with BIC and TTX demonstrated that developmental KCC2 up-regulation and GABA functional switch are independent of GABA_A_ receptor activation, which is in agreement with previous studies [27,45]. What may be responsible for KCC2 up-regulation during neuronal development? Our new findings suggest that KCC2 may be regulated by NL2, a cell adhesion molecule that expresses early during development. Indeed, we found that NL2 expression precedes that of KCC2 *in vivo* in early postnatal period, consistent with previous studies reporting that NL2 expression was first detected at embryonic day 16 while KCC2 was first detected at postnatal day 1 [14,24].

Our discovery of the NL2 regulation of KCC2 makes it easy to connect some interesting findings previously reported by different labs that are seemingly unrelated. For example, overexpression of NL2 in cerebellar granule cells has been shown to accelerate GABAergic synapse maturation by promoting the switch of GABA_A_ receptor α3 subunit to α1 subunit during early development [41]. Interestingly enough, overexpression of KCC2 was also found to accelerate this switch in cerebellar granule cells [46]. Our finding that NL2 regulates KCC2 may explain why NL2 and KCC2 both showed similar functions in promoting α subunit switch, although other links may also be possible. Therefore, NL2 is a chief conductor in orchestrating a variety of GABA functions including GABAergic synapse formation, GABA functional switch, and GABA_A_ receptor maturation.

### Neuroligin-2 regulates glutamatergic synapses through KCC2

Given the fact that NL2 is mainly localized at GABAergic synapses [14], it is initially puzzling that knockdown of NL2 not only reduced GABAergic synapses, but also reduced glutamatergic synapses. The original report using the same NL2shRNA also showed reductions in both glutamatergic and GABAergic synapses [15]. Interestingly, NL2 overexpression increased both glutamatergic and GABAergic synapse formation [15,17]. On the other hand, it has been shown that GABAergic, but not glutamatergic, transmission was decreased in NL2 knockout mice [42]. Such discrepancy between *in vitro* and *in vivo* data regarding the role of NL2 at glutamatergic synapses could be due to the difference between global knockout and shRNA-mediated knockdown. A recent study demonstrated that the transcellular differences in the relative amounts of NL1, rather than the absolute NL1 amount, governs the number of glutamatergic synapses *in vivo*[47]. Nevertheless, how NL2 might regulate glutamatergic synapses is not very clear.

KCC2 has been found to regulate dendritic spines. Specifically, Rivera and colleagues first reported that neurons from KCC2 knockout mice showed abnormally long dendritic protrusions and low frequency of mEPSCs [29]. Similarly, knockdown of KCC2 in developing neurons decreased the frequency of glutamatergic events [28]. KCC2 has also been shown to regulate the diffusion of AMPA receptors in dendritic spines [28]. Here, we demonstrated that NL2 regulates KCC2, raising a potential link between NL2 and glutamatergic synapses through KCC2. Indeed, overexpression of KCC2 together with NL2shRNA was able to rescue decreased glutamatergic synapses induced by NL2 knockdown, suggesting that KCC2 is likely the mediator of NL2 regulation of glutamatergic synapses. Therefore, NL2 not only regulates GABAergic synapses, but also regulates glutamatergic synapses through regulating KCC2, making NL2 a central player in balancing GABAergic and glutamatergic functions in the brain.

## Conclusion

To conclude, our data discovered a novel function of NL2 in regulating KCC2 and consequently affecting GABA functional switch as well as glutamatergic synapses. This finding extends the function of NL2 beyond its classical role in cell adhesion, and put NL2 in a central position in coordinating GABAergic synaptogenesis with GABA functional switch, and in balancing GABAergic and glutamatergic functions. We propose that NL2 may function as a master regulator of the delicate excitation-inhibition balance in the brain, and NL2 may be a novel drug target for developing the next generation of antipsychotic drugs.

## Materials and methods

### Cell culture

Primary mouse cortical neurons were cultured as previously described [11]. Briefly, cerebral cortices of newborn C57BL/6 mice of either sex were dissociated and plated on a monolayer of cortical astrocytes at a density of 8,000-12,000 cells/cm^2^ in 24-well plates. Culture medium contained MEM (500 ml, Invitrogen), 5% FBS (HyClone), 10 ml B-27 supplement (Invitrogen), 100 mg NaHCO_3_, 20 mM D-glucose, 2 mM Glutamax (Invitrogen), and 25 units/ml penicillin/streptomycin. Neurons were maintained at 37°C in a 5% CO_2_-humidified incubator. All experiments were repeated in at least three independent cultures.

### Transfection

Calcium-phosphate transfection in cultured neurons was performed similar to a protocol developed in our laboratory [48]. Plasmid at 1 μg each was used for transfection per well in a 24-well plate. HEK 293T cells were transfected using polyethylenimine (Polysciences). GFP or mCherry was coexpressed to identify transfected cells.

### Plasmids

NLmiR with IRES GFP or mCherry, HA-tagged shRNA-proof mouse NL1* and rat NL2* with IRES mCherry were generously provided by Dr. Roger Nicoll (University of California at San Francisco, San Francisco, CA). HA-tagged WT mouse NL2 and EGFP-NL2shRNA were provided by Dr. Peter Scheiffele (University of Basel, Basel, Switzerland). A non-tagged NL2shRNA was generated by cutting off EGFP with restriction enzymes. NLmiR and NL2shRNA have been characterized previously [15,22]. The NL2 target sequence of NL2shRNA and NLmiR is identical (ATGGAGCAAGTTCAACAGCAA) and conserved in mouse and rat. Rat KCC2 (pIRES2-EGFP) was provided by Dr. Yun Wang (Fudan University, Shanghai, China). mCherry (pEGFP-C1) was provided by Dr. Yingwei Mao (Pennsylvania State University, University Park, PA).

### Immunostaining and imaging analysis

Neurons were fixed in 4% paraformaldehyde for 8 min, permeabilized with 0.2% Triton X-100 for 5 min, and blocked with 5% normal donkey/goat serum for 30 min. Primary antibodies in blocking solution were incubated overnight at 4°C. Dylight-conjugated secondary antibodies (Jackson ImmunoResearch) were incubated at room temperature for 45 min. Following antibodies were used: KCC2 (07–432, Millipore), HA (sc-7392, Santa Cruz Biotechnology), MAP2 (ab5392, Abcam), NKCC1 (T4, Developmental Studies Hybridoma Bank), vGlut1 (135302, Synaptic Systems), GFP (ab13970, Abcam). Confocal images were collected on an Olympus FV1000 confocal microscope. For the quantification of HA, KCC2, and NKCC1 immunostaining, neuronal soma was selected and the mean intensity (0–255) was analyzed by ImageJ software. For spine density analysis, two secondary dendritic segments of 20 μm each were analyzed per neuron.

### Electrophysiology

The electrophysiological experiments were performed as previously described [21,49]. Briefly, Multiclamp 700A amplifier and pClamp software (Molecular Devices) were used for acquiring data (sampling at 10 kHz and filtered at 1 kHz). Neurons were continually perfused with bath solution (in mM): 128 NaCl, 30 glucose, 25 HEPES, 5 KCl, 2 CaCl_2_ and 1 MgCl_2_ (320 mOsm, adjusted to pH 7.3 with NaOH). Pipette solution contained (in mM): 147 KCl, 5 Na_2_-phosphocreatine, 2 EGTA, 10 HEPES, 2 MgATP, 0.3 Na_2_GTP (300 mOsm adjusted to pH 7.3 with KOH). Gramicidin (40 μg/ml, Sigma) was included in the pipette solution for perforated patch recording [26]. A Picospritzer (Parker Instrumentation) was used to eject GABA directly to neuronal soma through a fine pipette (~2 μm tip). In whole-cell patch clamp mode (holding at -70 mV), mEPSCs were recorded in the presence of TTX (0.5 μM) and BIC (20 μM); mIPSCs were recorded in the presence of TTX (0.5 μM) and DNQX (10 μM).

### Immunoblot

HEK 293T cells in 6-well plates were transfected using polyethylenimine and total protein lysate was harvested after 2–3 days in lysis buffer (20 mM HEPES, 1% Triton X-100, 0.1 mM EDTA, 2 mM CaCl_2_, 1mM MgCl_2_ and 50 mM NaCl with PMSF, protease and phosphatase inhibitors, pH 7.3 with NaOH). For cultured neurons, 2 wells from a 24-well plate were lysised at each time point. For mouse cortical proteins, cortices were dissected out and homogenized. After 2 hr rotation at 4°C, supernatant was harvested by centrifugation (12,000 g, 30 min). Protein concentration was measured by Bradford Protein Assay Kit (Thermo Scientific Pierce Protein Biology Products). Samples were incubated with NuPAGE LDS sample buffer and 1% β-mercaptoethanol at 95°C (for NL2) or 50°C (for KCC2) for 15 min before resolved in 10% SDS-PAGE and transferred to PVDF membrane. Primary antibodies including rabbit anti-KCC2 (07–432, Millipore), rabbit anti-NL2 (129202, Synaptic Systems), mouse anti-HA (sc-7392, Santa Cruz Biotechnology) and mouse anti-actin (612656, BD Transduction Laboratories) together with HRP-conjugated secondary antibodies (Abcam) were used. Immunoblot band intensities were measured with ImageJ software.

### Co-immunoprecipitation

HEK 293T cells in 10-cm dish were transfected and total protein lysate was harvested after 2 days in Pierece IP buffer (Thermo Scientific) with protease and phosphatase inhibitors. Protein lysate was pre-cleaned by incubation with Dynabeads M-280 sheep anti-mouse IgG (11201D, Invitrogen) for 2 hr at 4°C. About 2 mg protein lysate was then incubated with Dynabeads and 2 μg normal mouse IgG (PP54, Millipore) or mouse anti-HA antibodies overnight at 4°C. After washing with IP buffer and PBS, the immunoprecipitated proteins were eluted by NuPAGE LDS sample buffer (Invitrogen). Protein lysate from 2–3 month old mouse brain was processed following the same protocol with Dynabeads M-280 sheep anti-rabbit IgG (11203D, Invitrogen). Normal rabbit IgG (PP64, Millipore) was used as control.

### Calcium imaging

Cells were incubated in 2.5 μM Fura-2 AM (Invitrogen) for 45 min at 37°C and washed for 15 min in bath solution. Coverslips were transferred to a perfusion chamber mounted on a Nikon TE-2000-S inverted microscope with a 20× objective and imaged with a 340/380 nm transmittance filter set (Chroma Technology). SimplePCI (HCImage, Hamamatsu) was used to measure the ratio of 340/380 fluorescence signal in neuronal soma. Sister coverslips from 3–4 independent cultures were taken for imaging at 3–4 time points. mCherry was coexpressed to identify transfected cells in all calcium imaging experiments. All recordings were done in the presence of DNQX (10 μM) to block AMPA receptor activations. The threshold of a significant Ca^2+^ response was set as 10 times of baseline standard deviation.

### Statistical analysis

Unpaired Student’s *t*-test was used for comparisons between two groups. One-way ANOVA with Bonferroni multiple comparisons was used for comparisons between multiple groups. Two-way ANOVA with Bonferroni multiple comparisons was used for comparisons between multiple time points and groups. GraphPad Prism (GraphPad Software) was used for all statistical analysis. Data were shown as mean ± standard error in all bar graphs.

## Abbreviations

NL2: Neuroligin-2; KCC2: Potassium-chloride transporter 2; NL2shRNA: Short hairpin RNA targeting NL2; NLmiR: Short hairpin RNA targeting NL1-3; BIC: Bicuculline; TTX: Tetrodotoxin; DIV: Days in vitro; EGABA: GABA equilibrium potential.

## Competing interest

The authors declare that they have no competing interests.

## Authors’ contributions

CS and LZ performed the experiments; CS, LZ, and GC analyzed the data; CS and GC designed the experiments and wrote the paper. All authors read and approved the final manuscript.
